# Wetland soil organic carbon balance is reversed by old carbon and iron oxide additions

**DOI:** 10.3389/fmicb.2023.1327265

**Published:** 2024-01-08

**Authors:** Bingbo Ni, Xiaofei Yu, Xun Duan, Yuanchun Zou

**Affiliations:** ^1^State Key Laboratory of Black Soils Conservation and Utilization and Heilongjiang Xingkai Lake Wetland Ecosystem National Observation and Research Station and Key Laboratory of Wetland Ecology and Environment and Jilin Provincial Joint Key Laboratory of Changbai Mountain Wetland and Ecology, Northeast Institute of Geography and Agroecology, Chinese Academy of Sciences, Changchun, China; ^2^University of Chinese Academy of Sciences, Beijing, China; ^3^State Environmental Protection Key Laboratory for Wetland Conservation and Vegetation Restoration and Jilin Provincial Key Laboratory of Ecological Restoration and Ecosystem Management and Key Laboratory of Vegetation Ecology of Ministry of Education, School of Environment, Northeast Normal University, Changchun, China

**Keywords:** Iron-bound organic carbon, organic carbon dynamics, trade-off effects, freshwater mire, saline-alkaline marsh

## Abstract

Iron (Fe) oxides can stabilize organic carbon (OC) through adsorption and co-precipitation, while microbial Fe reduction can disrupt Fe-bound OC (Fe-OC) and further increase OC mineralization. The net effects of OC preservation and mineralization mediated by Fe oxides are still unclear, especially for old carbon (formed from plant litters over millions of years) and crystalline Fe oxides. Accelerating the recovery of wetland carbon sinks is critical for mitigating climate change and achieving carbon neutrality. Quantifying the net effect of Fe-mediated OC mineralization and preservation is vital for understanding the role of crystalline Fe oxides in carbon cycling and promoting the recovery of soil carbon sinks. Here, we explored the OC balances mediated by hematite (Hem) and lignite addition (Lig) to freshwater wetland (FW, rich in C and Fe) and saline-alkaline wetland (SW, poor in C and Fe) soil slurries, incubated under anaerobic conditions. Results showed that Lig caused net OC accumulation (FW: 5.9 ± 3.6 mg g^−1^; SW: 8.3 ± 3.2 mg g^−1^), while Hem caused dramatic OC loss, particularly in the FW soils. Hem inhibited microbial Fe(III) reduction by decreasing the relative abundance of Fe respiration reducers, while substantially enhancing OC mineralization through the shift in the microbial community structure of FW soils. Lig resulted in carbon emission, but its contribution to preservation by the formation of Fe-OC was far higher than that which caused OC loss. We concluded that crystalline Fe oxide addition solely favored the increase of OC mineralization by adjusting the microbial community structure, while old carbon enriched with an aromatic and alkyl promoted Fe-OC formation and further increased OC persistence. Our findings could be employed for wetland restoration, particularly for the recovery of soil carbon sinks.

## 1 Introduction

Wetlands cover only approximately 8 percent of the total land area on Earth but store between 29 and 45 percent of the terrestrial organic carbon (C) (Chen et al., 2021). Over the last three centuries, about 21 percent of global wetlands have been lost due to climate change and anthropogenic disturbances, such as wetland reclamation, drainage, and human-induced fires (Fluet-Chouinard et al., 2023). Consequently, the ongoing decrease in carbon sinks and the continuous increase in carbon emissions, further exacerbate climate change (Xiao et al., 2019). Therefore, accelerating the recovery of wetland carbon sinks is critical for mitigating climate change and achieving carbon neutrality.

The strong interlinked association between minerals and organic carbon (OC) acts as a key factor in the long-term storage of soil OC (SOC) (Lalonde et al., 2012; Rasmussen et al., 2018; Duan et al., 2020). Reactive (Iron [Fe]- and aluminum-bearing) minerals in soils that are retained in OC contribute 3–72% of the SOC, depending on effective moisture at the global scale (Kramer and Chadwick, 2018). Fe oxides protect OC through adsorption or co-precipitation, and the binding mechanisms can be divided by C:Fe molar ratios. C:Fe molar ratios of <1 and > 6 showed Fe-C complexes derived by adsorption and co-precipitation, respectively (Wagai and Mayer, 2007; Lalonde et al., 2012; Chen et al., 2020). The initial coupled Fe-C association can serve as nuclei for soil aggregate formation, further isolate OC from microbes and restrict oxygen diffusion against decomposition (Kögel-Knabner et al., 2008; Kleber et al., 2015). Therefore, Fe-bound OC (OC-Fe) was considered to persist for centuries or millennia (Lalonde et al., 2012).

Common Fe oxides are redox-sensitive, and play the role of a “rusty sink” for carbon sequestration and act as an electronic acceptor in microbially mediated dissimilatory Fe reduction (DIR), which is a crucial mechanism for Fe dissolution and OC mobilization (Pan et al., 2016; Poggenburg et al., 2016). Geobacter and Shewanella are key Fe-reducing bacteria that carry out electron transfer by functioning as biological nanowires to the Fe(III) mineral surface (Adhikari et al., 2017; Kappler et al., 2021). Moreover, Fe oxides can also shape the soil microbial community by providing niche-specific conditions and further influence OC dynamics (Jeewani et al., 2021b). Fast-growing bacteria (e.g., Proteobacteria, Actinobacteria, and Mortierellomycota) preferentially colonize Fe-rich soils (Converse et al., 2015; Jeewani et al., 2021b). lignite is an old C that may be suitable as a soil conditioner because it is a rich source of organic C, relatively abundant and inexpensive. Lignite is formed from plant remains via coalification under heat and pressure (Kim Thi Tran et al., 2015). Compared to plant litter, microbial use for lignite is weaker (Rumpel, 2004), and it may be easier to accumulate in soil.

Recent studies have reported that the “new carbon” (e.g., root exudates and microbial metabolites) could also disrupt the Fe-bound OC (Fe-OC) associations. by mobilizing Fe-OC directly or altering microbial community (Clarholm et al., 2015; Keiluweit et al., 2015). Glucose can also enhance mineral-associated OC destabilization and mineralization by increasing enzyme activity, such as cellulase and chitinase (Jilling et al., 2021). However, little is known about the effect of “old carbon” associations. A previous study showed that the stability of ‘old carbon’ that formed over millions of years in deep soil layers is maintained without a fresh OC supply (Fontaine et al., 2007); however, there is also evidence that old but highly bioreactive OC could be converted to CO_2_ by microbial respiration (McCallister and Del Giorgio, 2012). Therefore, mineral-bound OC, either new or old, is not an inert or passive pool, but a dynamic one that can potentially provide a source of carbon for microbes or plants (Kleber et al., 2021; Li et al., 2021). Considering the duality of Fe oxides in OC (Song et al., 2022), quantifying the net effect of Fe-mediated OC mineralization and preservation is vital for understanding the role of crystalline Fe oxides in carbon cycling and promoting the recovery of soil carbon sinks.

We selected two different Fe and OC content wetland soils, that is, freshwater wetland (FW; rich in Fe and OC) and saline-alkaline wetland (SW; poor in Fe and OC), to explore the trade-off in Fe oxide-mediated OC preservation and mineralization in a 63-day incubation experiment. We added exogenous Fe oxides (Hem) and OC (Lig) to the incubation experiment to test whether the formation of Fe-C complexes is limited by the sorbent or substrate. we hypothesize that (i) hematite (Hem) and lignite (Lig) addition can increase Fe-OC formation because Hem, a crystalline Fe oxide, is difficult to utilize by Fe-reducing bacteria, and the turnover of OC-associated crystalline Fe oxides is slower than their retention by short-range-order Fe oxides; (ii) Hem regulates OC mineralization by shifting the microbial community structure; and (iii) Hem and Lig cause OC preservation effects that are higher than OC mineralization, and the materials could be used to accelerate the recovery of wetland soil carbon sinks.

## 2 Materials and methods

### 2.1 Soil, Hem, and Lig preparation

Soil samples were collected from the Honghe National Nature Reserve (HNNR, 133°34′–133°46′E, 47°42′–47°52′N) in Heilongjiang Province (Lou et al., 2020), and the Momoge National Nature Reserve (MNNR, 122°27′–124°4′E, 45°45′–46°10′N) in Jilin Province of Northeast China (An et al., 2019). which belonged to the freshwater and saline-alkaline wetlands, respectively. The soil from the freshwater wetland contained 43.2 g kg^−1^ organic C, 3.3 g kg^−1^ total N. The soil from the saline-alkaline wetland had 16.2 g kg^−1^ organic C, 1.2 g kg^−1^ total N. Soil pH of the two wetlands was 6.54 and 9.02, respectively. The average annual temperature and precipitation were *ca.* 1.9°C and 585 mm in HNNR and 4.2°C and 392 mm in MNNR, respectively. The dominant plants growing at HNNR and MNNR were *Calamagrostis angustifolia* and *Scirpus planiculmis*, respectively.

For each sample, an upper-layer soil core (0–20 cm) was collected after the plant debris was removed. Fifteen cores from each wetland were randomly collected and gently homogenized, stored in a portable refrigerator after sealing, and transported to the laboratory. A part of the soil was used to analyze the physical and chemical properties stored at 4°C, and the remaining soils were freeze-dried and sieved (<0.2 mm) before incubation.

Hem was synthesized as suggested by Christl et al. (2012). Briefly, 100 mL of 2 M FeCl_3_ solution was slowly added to 100 mL of 5.4 M NaOH solution for 5 min. The obtained colloids were aged at 101°C for 8 h and cooled to room temperature (20°C) after aging. The suspension was transferred to centrifuge tubes and centrifuged at 3,000 g for 25 min using ultrapure water to remove salt ions (5 uS/cm^1^). The purified precipitate was freeze-dried, sieved (<0.2 mm), and stored at 4°C for use.

Lig was provided by the Shenhua Guoneng Baoqing Coal Electricity Chemical Co., located in Baoqing County of Heilongjiang Province. The air-dried and sieved (<0.2 mm) Lig was stored at 4°C for further use. The Lig was composed of 3.2% polysaccharides, 9.0% aliphatics, 38.9% alkyl, 29.3% aromatics, 8.7% N-containing compounds, and 11.0% phenols analyzed by pyrolysis (Frontier Lab PY-3030, Japan) and chromatography-mass spectrometry (Agilent 5,975 T LTM-GC/MSD, United States).

### 2.2 Experiment design

To avoid the photochemical reactions associated with Fe oxides, we performed an anaerobic incubation experiment using brown bottles. Before incubation, all bottles were repeatedly washed with ultrapure water and sterilized at 108°C for 4 h. The prepared soils were mixed with sterile deionized water at a ratio of 1:1 (20 g freeze-dried soil added to 20 mL sterile deionized water) to form soil slurries. The average contents of total Fe oxides and OC in the topsoil layers of SW are significantly lower than those in FW. Consequently, Hem and Lig were referred to the Fe and OC contents of the SW. Hem was added at a concentration of 0.4 mmol of Fe g^−1^ dry soil, and Lig was added at a concentration of 2.9 mmol of Fe g^−1^ dry soil. A total of 40 mL of the mixed soil slurry was transferred to 100 mL brown bottles, and four treatments were set up for each wetland soil, (i) Hem + soil slurry, (ii) Lig + soil slurry, (iii) Hem + Lig + soil slurry, and (iv) soil slurry (CK). The bottles were sealed with a rubber stopper and covered with aluminum plastic cover. To control the exchange of air, a three-way valve was plugged into the rubber stopper, which was convenient for the gas samples. Each treatment was performed in 12 duplicates and incubated at 25°C in an anaerobic glove box for 63 days.

Half of the sample was used destructively to measure Fe(II) and Fe(III) at 1, 4, 7, 14, 21, 35, 49, and 63 d. On the same days, the gas samples were collected through a three-way valve using a plastic syringe (50 mL) to measure the CO_2_ and CH_4_ concentrations. We also measured the soil pH at 4, 21, and 63 d during anaerobic incubation. Specific Fe oxides and co-dissolved OC, SOC chemical composition, and microbial community structure were analyzed post-incubation.

### 2.3 Soil chemical properties and gas analyses

Soil pH was measured using a pH microelectrode (Unisense, Denmark) in an anaerobic glove box. The SOC in air-dried and acid-flushed soils was determined using an elemental analyzer (Elementer Vario EL III, Germany). Dissolved organic carbon (DOC) was extracted at a ratio of 1:5 (w/v), shaken for 16 h, and centrifuged at 3,000 g. Following filtration using 0.45 μm nylon filters, the OC contents were determined using a TOC analyzer (Shimadzu TOC-VCPH, Japan). Fe(II) was extracted using hydrochloric acid (HCl) and measured using the 1,10-phenanthroline colorimetric method on a UV spectrophotometer (Shimadzu UV-2500, Japan) (Kostka and Luther, 1994).

The concentrations of CO_2_ and CH_4_ were measured using gas chromatography (Agilent 7,890 B GC, USA). The production rates were calculated as follows:


(1) $$F=\rho \times V/W\times \Delta_{c}/\Delta_{t}\times 273/T\times \alpha$$

Where F is the production rate of each treatment (m^3^ C g^−1^ d^−1^); ρ is the gas density (1.98 kg m^−3^ for CO_2_ and 0.714 kg m^−3^ for CH_4_); *V* is the void volume of the incubation bottle (m^3^); W is the weight of soil (g); Δc/Δt is the ratio of change in the gas concentration per unit time during the sampling period (μg g^−1^ *d*^−1^); *T* is the incubation temperature (*K*); α is a conversion factor for CO_2_ to C (=12/44) and CH_4_ (=12/16).

The cumulative emissions of CO_2_ and CH_4_ were calculated as follows:


(2) $$M=\sum \left(F_{i+1}+F_{i}\right)/2\times \left(t_{i+1}-t_{i}\right)$$

Where M is the cumulative emission of each treatment (mg C g^−1^); F is the production rate of each treatment (m^3^ C g^−1^ *d*^−1^); t is the sample time (*d*).

A sequential selective dissolution method was applied to quantify Fe oxides and their associated OC contents using three chemical extractions to avoid overlap. First, Here, we extracted the organically complex Fe and their bound OC from 0.5 g freeze-dried soil (milled <200 μm) using 30 mL 0.1 M sodium pyrophosphate(pH = 10). After 16 h of shaking and 40 min high-speed centrifugation, an aliquot of the extract was immediately used for measuring dissolved OC and Fe using a TOC analyzer and inductively coupled plasma atomic emission spectroscopy (Shimadzu ICPS-7500, Japan), respectively. Second, the soil residue was repeatedly flushed with ultrapure water to remove the remaining supernatant. The short-range-order Fe oxides and their bound OC were then determined using 30 mL of acidified hydroxylamine (pH = 2). After shaking for 30 min in the dark and high-speed centrifugation for 40 min, the dissolved OC and Fe in the supernatant were measured. Finally, after flushing, 30 mL of 57.4 mM sodium dithionite was added to the soil residue, shaken for 16 h, and then centrifuged. The extracted supernatant was stored in other clear tubes. Further, 0.05 M HCl was added to the soil residue. After shaking at 100 rpm for 1 h and centrifuging at 3,000 g for 30 min, the supernatant was mixed with the supernatant extracted using sodium dithionite, and the mixed supernatant was sampled for the analysis of crystalline Fe oxides and their bound OC (Coward et al., 2017; Heckman et al., 2018; Wagai et al., 2020).

### 2.4 DNA extraction and high-throughput sequencing analysis

A fresh soil sample (0.5 g) was used to extract DNA after anaerobic incubation using a power soil DNA isolation kit (Mo Bio Laboratories, Carlsbad, CA, United States) according to the manufacturer’s protocol. The extracted DNA was stored at −80°C until further analysis.

The extracted DNA was dissolved in 50 μL TE buffer, and the NanoDrop 2000 (Thermo Scientific, Willmington, DE, USA) system was used to quantify the concentrations of DNA. The bacterial regions V4–V5 of the 16S rRNA gene were PCR-amplified using the forward primer 515F (5′-GTGCCAGCMGCCGCGGTAA-3′), and the reverse primer 907R (5′-CCGTCAATTCMTTTRAGTTT-3′). The fungal region, ITS1, was amplified by PCR using the 5′-CTTGGTCATTTAGAGGAAAAGTAA-3′ forward primer and 5′-GCTGCGTTCTTCATCGATGC-3′ reverse primer. After the samples were purified and pooled, high-throughput sequencing analysis was performed using the Illumina HiSeq 2,500 platform (2 × 250 paired-ends) at the Center for Genetic and Genomic Analysis, GENESKY Biotechnologies Inc. (Shanghai, China).

### 2.5 Statistical analyses

Statistical analyses were performed and figures were prepared using R software version 3.6.3.^1^ We determined the difference in OC preservation, mineralization and balance among soils using the “aov” function, and then the “TukeyHSD” function was used for post-hoc analysis. The “lm” function was used to evaluate the linear regression relationship between Fe oxides and their co-dissolved OC. Microbial α and β diversity were analyzed using “picante” and “vegan” packages (Dixon, 2003; Kembel et al., 2010).

We performed random forest regression analysis using the function “random forest” of the “randomForest” package to evaluate the contribution of bacterial phyla (Actinobacteria, Bacteroidetes, Firmicutes, Acidobacteria, Chloroflexi, Planctomycetes, Proteobacteria, and Ignavibacteriae) and fungal phyla (Zygomycota, Chytridiomycota, Basidiomycota, and Ascomycota) to OC mineralization. The increase in the mean squared error (IncMSE, %) represents the relative importance of the response variable (Liaw and Wiener, 2002).

Piecewise structural equation modeling (Piecewise SEM) was performed in the “piecewiseSEM” package using the function “psem” to explore the drivers and pathways of OC mineralization and preservation. The pH, Fe oxides extracted by sodium pyrophosphate (Fe_PP_), Fe oxides extracted by acidified hydroxylamine (Fe_HH_), and biological factors (e.g., bacterial and fungal Shannon indices) were considered to influence the OC dynamics. Considering the non-significant relationship between Fe oxides extracted by dithionite-HCl (Fe_DH_) and OC extracted by dithionite-HCl (OC_DH_), we excluded this variable in Piecewise SEM. Fisher’s C test was used to assess model fitting, whereas a non-significant chi-square test (*p* > 0.05) indicated a good fit of the Piecewise SEM to the data. Akaike’s information criteria and Bayesian information criteria were used for model selection when more than two fitted Piecewise SEM models met the model evaluation (Lefcheck, 2016).

## 3 Results

### 3.1 OC preservation by Fe oxides

**Figure 1:**
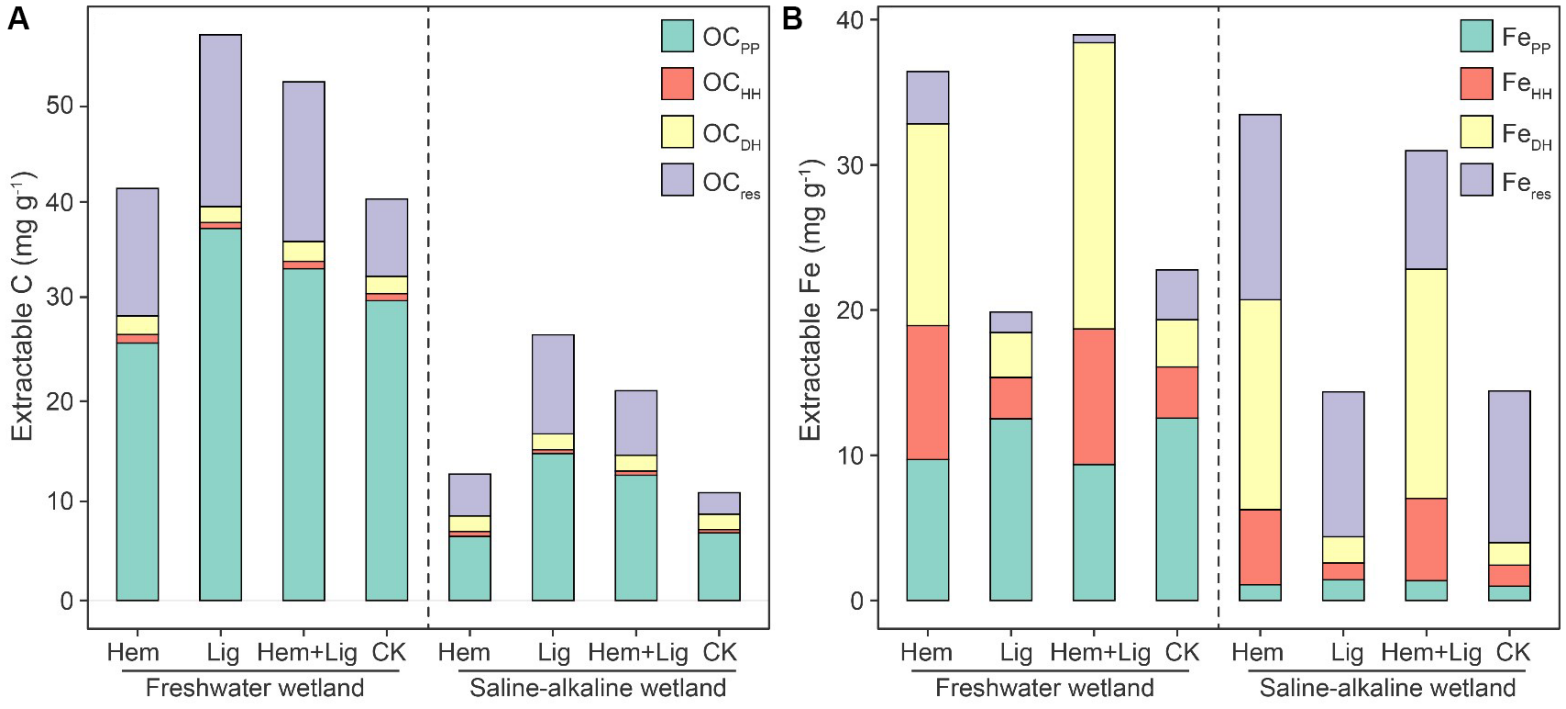
Changes of extracted organic carbon (OC), **(A)** and iron (Fe), **(B)** using sodium pyrophosphate (PP), acidified hydroxylamine (HH) and dithionite-hydrochloric acid (DH) in freshwater and saline-alkaline wetlands soils responding to hematite addition (Hem), lignite addition (Lig) and both additions (Hem+Lig) respectively (*n* = 6). OCpp, OCHH and OCDH were organic carbon extracted by PP, HH, and DH respectively; Fepp, FeHH, and FeDH were Fe oxides extracted by PP, HH, and DH, respectively.

Surprisingly, OC extracted by sodium pyrophosphate (OC_PP_) accounted for 50.91–74.79% of SOC in FW and SW. Compared to SW, greater OC_PP_ content was found in FW due to its higher Fe_PP_ content (*p* < 0.05, Figure 1). Compared to OC_PP_, OC extracted by acidified hydroxylamine (OC_HH_) and OC_DH_ only contributed to less than 5% of SOC, although Fe_HH_ and Fe_DH_ were not low in both wetland soils (Figure 1).

**Figure 2:**
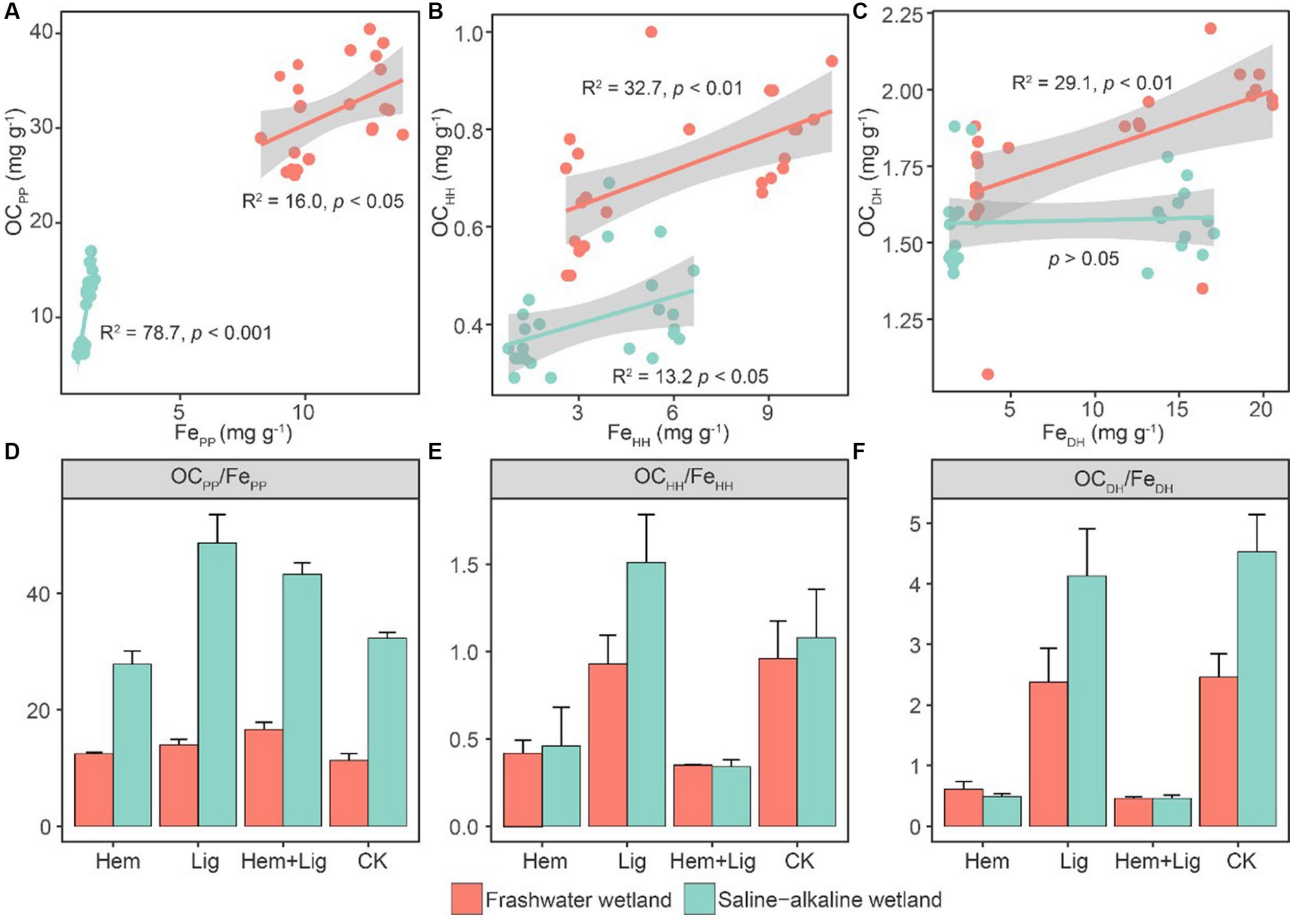
Relationships between OCPP and FePP **(A)**, OCHH and FeHH **(B)**, and OCDH and FeDH **(C)** and their ratios **(D−F)** responding to Hem and Lig in freshwater (red circle) and saline-alkaline (green circle) wetlands soils, respectively. Regression lines are only shown for significant relationships (*n* = 6, *p* < 0.05).

The Lig addition enhanced the OC_PP_ content in both wetland soils. The addition of Hem significantly increased the Fe_HH_ and Fe_DH_ contents in FW and SW (*p* < 0.05). However, its co-dissolved carbon increased to approximately 7 and 16% in FW soils amended with Hem and Hem + Lig, respectively. A highly positive correlation was observed between the Fe oxides and OC extracted using sodium pyrophosphate (Figure 2A). Fe_HH_ also showed a positive association with co-dissolved carbon. The positive relationship between OC_DH_ and Fe_DH_ only occurred in FW (Figures 2B,C). The C/Fe extracted using sodium pyrophosphate ranged from 11 to 48, which was significantly lower in Hem-treated soils compared to other treatments in both wetland soils (p < 0.05, Figure 2D). The C/Fe extracted by acidified hydroxylamine and dithionite-hydrochloric acid was higher in the Lig treatment, which ranged from 0.4–1.7 and 0.6–5.0 in FW and SW soils, respectively (Figures 2E,F).

### 3.2 Fe(II) production and carbon emission

**Figure 3:**
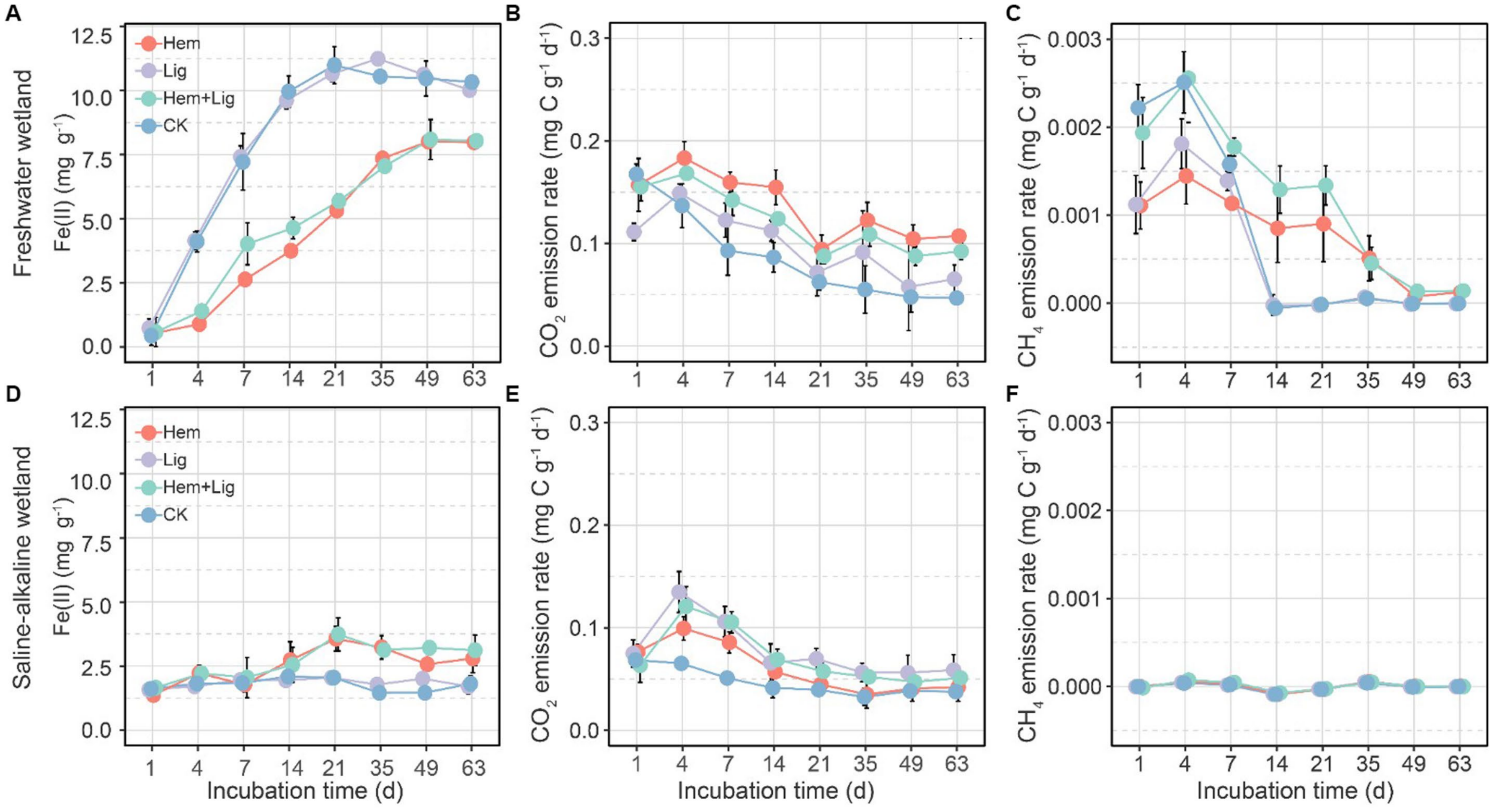
Fe(II) concentrations, CO2, and CH4 emission rates variations of freshwater **(A−C)** and saline-alkaline **(D−F)** wetlands soils response to hematite addition (Hem), lignite addition (Lig) and both additions (Hem + Lig), respectively, over the incubation period (*n* = 6).

Lig had a non-significant effect on Fe(II) produced in the two soils over the incubation period (Figures 3A,D), but it exerted a significant decrease in Hem FW soils (Figure 1A) and a slight increase in Hem SW soils (Figure 3D). Moreover, Fe(II) levels were higher in FW than in SW during the incubation period (Figures 3A,D).

The CO_2_ emission rates only increased in the first 4 days, gradually decreased on days 4 to 63 in each treatment of both soils, and were higher in FW soils than in SW soils (Figures 3B,E). Hem and Lig caused the highest CO_2_ emission rates in FW and SW soil, respectively (Figures 3B,E). Interestingly, CH_4_ emission rates of FW soils treated with Lig and CK showed a sharp reduction on days 4 to 14 (Figure 3C), but those of Hem soils decreased gradually (Figure 3C). Surprisingly, in the SW soils, CH_4_ emission rates in each treatment were maintained at *ca.* 0 (Figure 3F). The highest cumulative CO_2_ emissions occurred in the Hem of FW soil but not in the Lig of SW soil. Hem + Lig resulted in the highest cumulative CH_4_ emissions of FW soil, whereas no significant difference was found in all additions of SW soil.

### 3.3 OC preservation, mineralization, and balance

**Figure 4:**
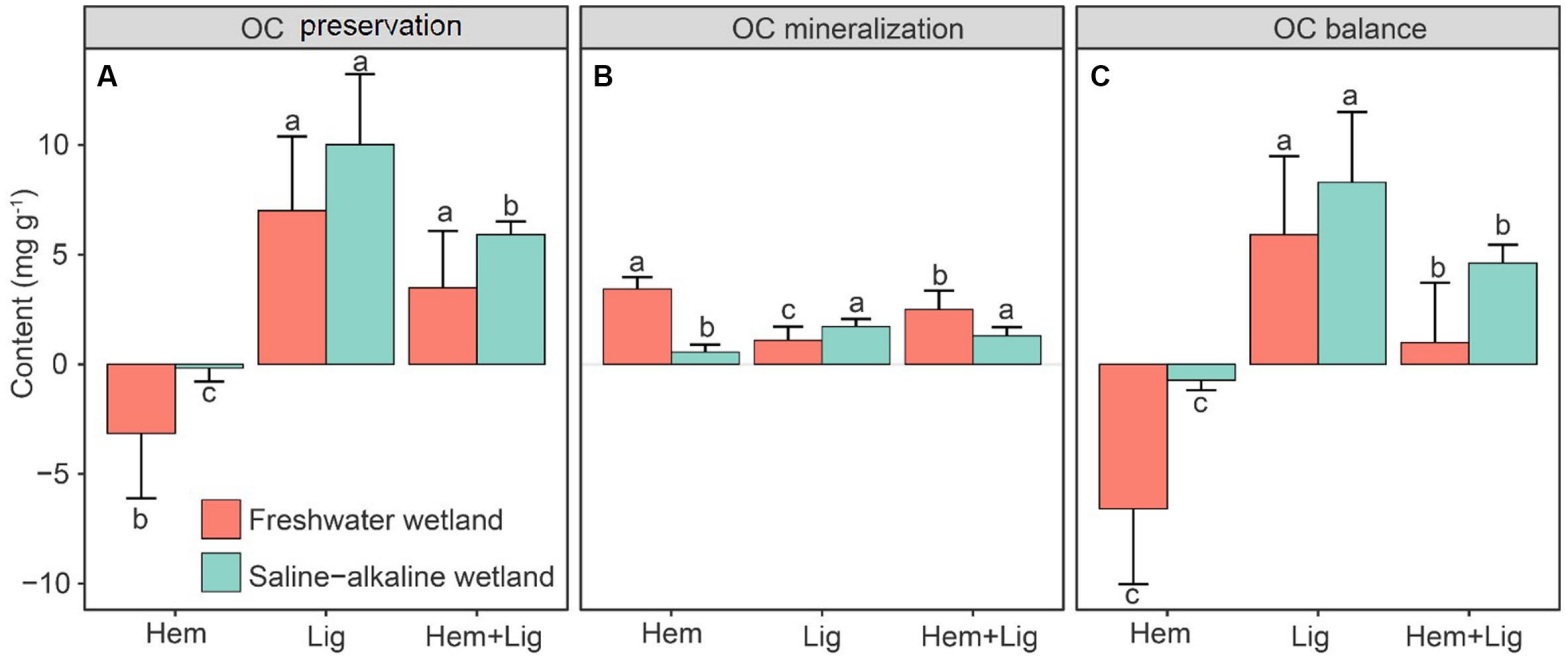
Organic carbon (OC) preservation by Fe oxides **(A)**, microbial mineralization, **(B)** and the balance (Fe mediated OC preservation vs. microbial mineralization) of the first two variables **(C)** in freshwater (red bars) and saline-alkaline (green bars) wetlands soils responding to Hem and/or Lig after 56 days of anaerobic incubation (*n* = 6).

The highest stabilized OC by Fe oxides in FW and SW occurred in Lig, followed by Lig + Hem. However, Hem had a negative effect on OC preservation (Figure 4A). The highest OC mineralization in FW was recorded after the addition of Hem, whereas the lowest OC mineralization was found in SW. Lig showed a significant increase in OC mineralization in the SW (*p* < 0.05, Figure 4B). According to the balance of OC preservation and mineralization, Lig caused net OC accumulation (FW: 5.9 ± 3.6 mg g^−1^; SW: 8.3 ± 3.2 mg g^−1^), but Hem resulted in dramatic OC loss, particularly in FW soils during the 63-day anaerobic incubation. In the Hem + Lig treatment, positive OC accumulation by Lig was higher than the negative effect induced by Hem (Figure 4C). Therefore, Lig + Lig showed a relatively low OC accumulation compared to Lig (Figure 4).

### 3.4 Microbial diversity and community structure

**Figure 5:**
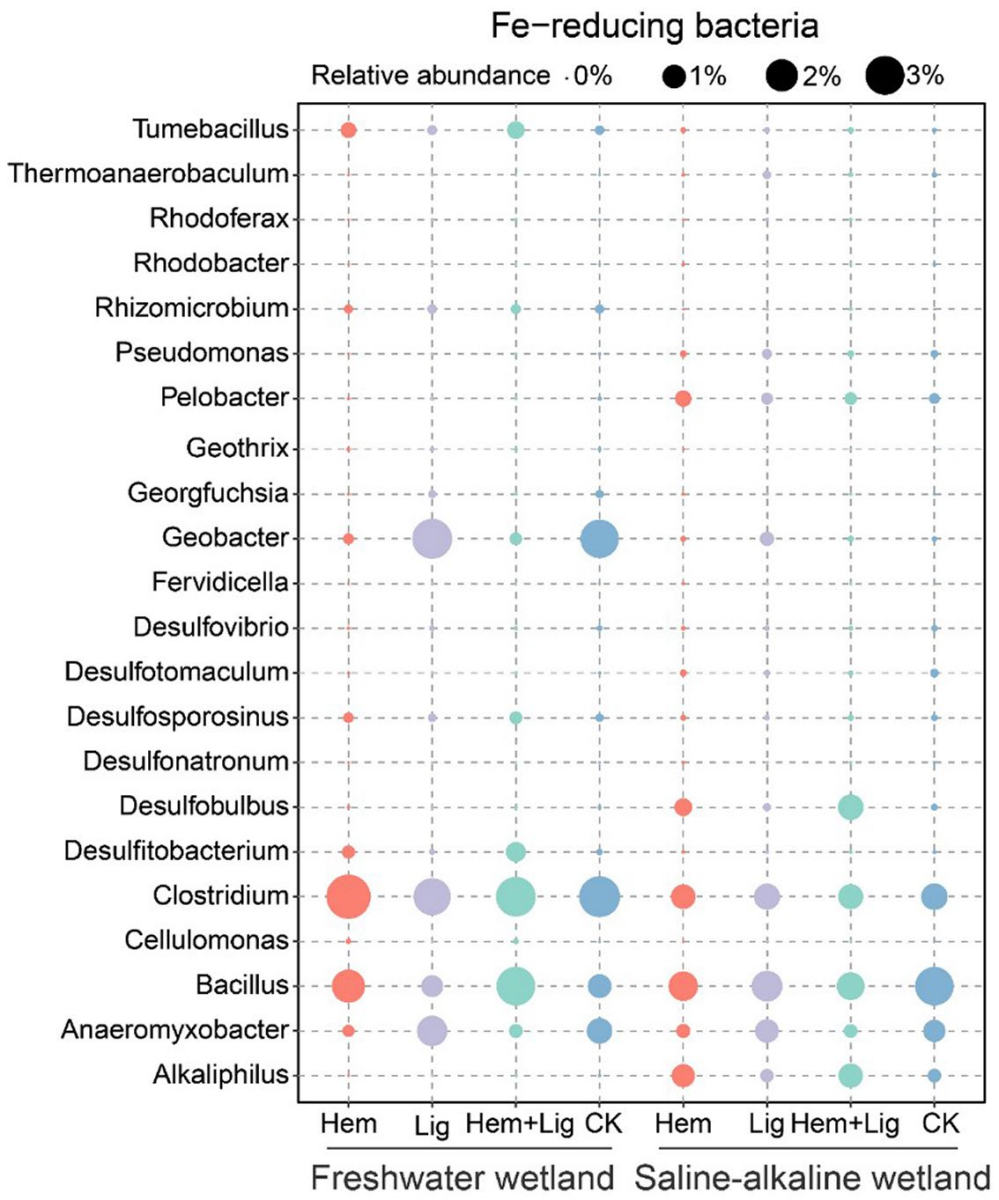
Principal coordinates analysis (PCoA) showed beta diversity based on Bray–Curtis dissimilarity of microbial community structure at the OUTs level in freshwater [**(A)**: bacteria, **(B)**: fungi] and saline-alkaline [**(D)**: bacteria, **(E)**: fungi] wetlands soils after 56 days of anaerobic incubation (*n* = 6). Relative abundance of the major bacterial **(C)** and fungal phylum **(F)** in freshwater and saline-alkaline wetlands soils (*n* = 6).

Lig resulted in the highest bacterial Shannon index in FW and SW soils (Supplementary Figure S2a). The highest fungal Shannon index of FW occurred in Hem + Lig, but that of SW occurred in Lig. Principal coordinates analysis showed that Hem and Lig shaped the bacterial and fungal communities of FW and SW at the (operational taxonomic unit) OUT level (Figures 5A,B,D,E). However, the higher variance of bacterial communities compared to that of fungal communities (FW: 60.52%; SW: 55.41%) was explained (FW: 21.21%; SW: 19.76%). Among the bacterial communities in FW, Hem + Lig exhibited patterns similar to those of Hem but not Lig (Figure 5A).

**Figure 6:**
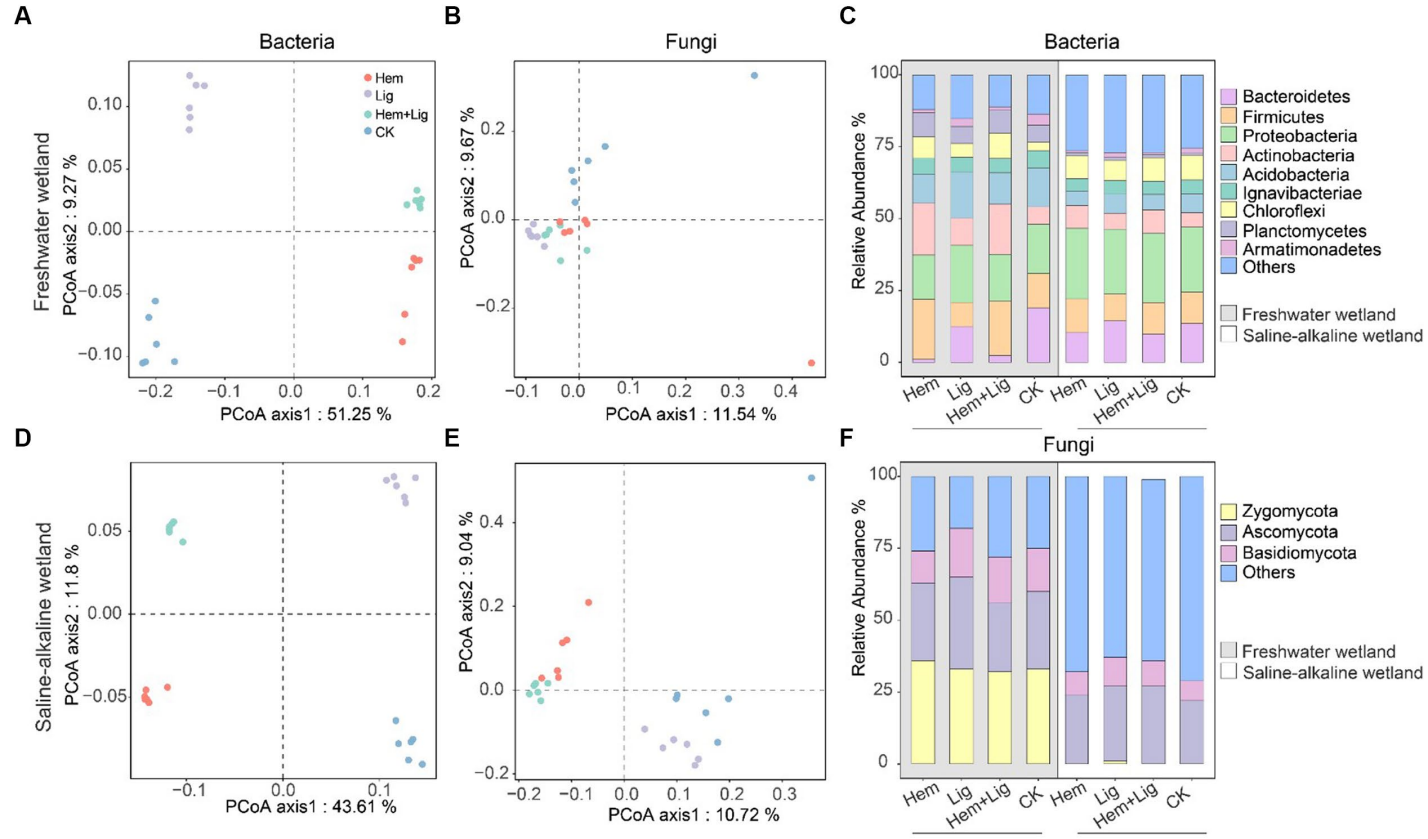
Fe-reducing bacteria at genus level of freshwater and saline-alkaline wetlands soils responding to Hem and Lig after 56 days of anaerobic incubation (*n* = 6).

The relative abundance of bacterial communities in FW at the phylum level showed distinct differences due to Hem and Lig (Figures 5C,F). Hem and Hem + Lig resulted in a dramatic decrease in the relative abundance of Acidobacteria and Bacteroidetes, and a significant increase in that of Firmicutes, Actinobacteria, and Chloroflexi (*p* < 0.05, Figure 5C). Among the bacterial phyla, Proteobacteria was dominant in each treatment of FW (Figure 5C). For the fungal phyla, the relative abundance of Zygomycota and Basidiomycota in FW was significantly higher than that in SW (*p* < 0.05, Figure 5F). Hem significantly decreased the relative abundance of Geobacter and Anaeromyxobacter in FW and SW soils (*p* < 0.05, Figure 6). The relative abundances of Alkaliphilus and Desulfobulbus in the SW soil dramatically increased because of Hem (Figure 6). For the Fe-reducing bacteria, Hem significantly decreased the relative abundance of Geobacter and Anaeromyxobacter in FW and SW soils (*p* < 0.05, Figure 6), but decreased the relative abundance of Bacillus. Hem increased Clostridium and Bacillus in FW, whereas the effect was absent in SW.The relative abundances of Alkaliphilus and Desulfobulbus in the SW soil dramatically increased because of Hem (Figure 6).

### 3.5 Associations between microbes and OC dynamics

Random forest regression analysis revealed that the bacterial phyla, including Actinobacteria, Bacteroidetes, Firmicutes, and Acidobacteria, were the main drivers of OC mineralization in FW (*R*^2^ = 73.49%, Supplementary Figure S1a). Firmicutes and Planctomycetes were the most important contributors to OC mineralization (*R*^2^ = 37.73%) in the SW (Figure 5B). The contribution of fungal phyla to OC mineralization was lower than that of bacteria, regardless of the wetland (Supplementary Figure S1).

**Figure 7:**
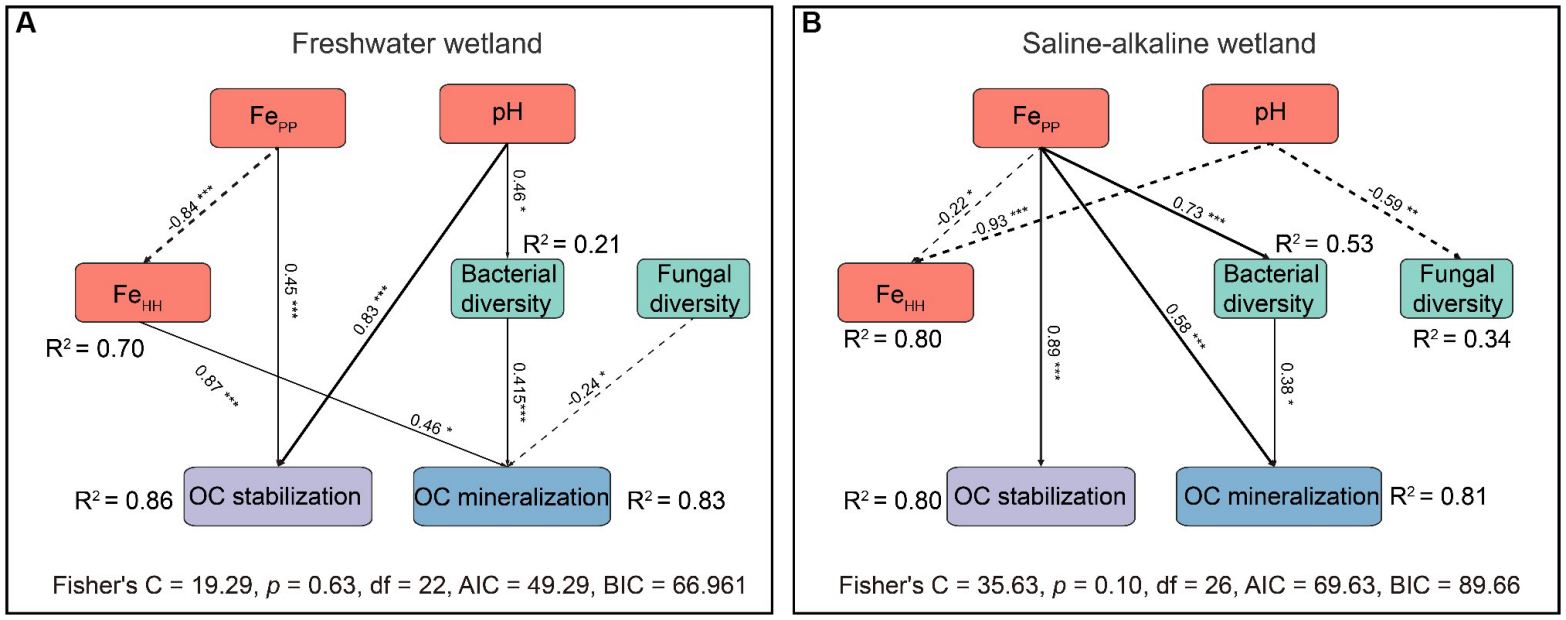
Piecewise structural equation modeling (Piecewise SEM) exploring the effects of pH, iron oxides, and microbial diversity on OC preservation and mineralization of freshwater **(A)** and saline-alkaline **(B)** wetlands soils. Solid lines indicate positive pathways, and dashed lines indicate negative pathways. R2 represents the explained proportion of the dependent variable. Asterisks represent the significance level of standardized pathway coefficients (*p < 0.05, **p < 0.01, and ***p < 0.001). FePP, organically complexed Fe oxides extracted by sodium pyrophosphate; FeHH, short-range-order Fe oxides extracted by acidified hydroxylamine. Bacterial and fungal diversities are represented by the Shannon index.

Piecewise SEM showed that chemical and biological factors explained more than 80% of the variance in OC preservation and mineralization in FW and SW (Figure 7). Interestingly, OC mineralization in FW and SW was regulated by chemical and biological factors, but OC preservation was only adjusted by chemical variables (Figure 7). pH and Fe_PP_ showed a dramatic positive relationship with OC preservation. Increasing pH, Fe_HH_, and bacterial diversity could promote OC mineralization in FW, whereas increasing fungal diversity and Fe_PP_ could suppress it. OC mineralization in SW had a strong positive association with Fe_PP_ and bacterial diversity. Fe_PP_ was the only contributor to OC preservation in SW.

## 4 Discussion

### 4.1 Effect of Fe oxides on OC preservation

We assessed the contribution of special Fe phases to OC preservation in wetland soils. Lig leads to substantial OC-Fe accumulation. Higher Fe_PP_ considerably promoted the complexation of OC with Fe oxides in FW and SW soils (*p* < 0.05, Figure 1). Similar findings were also reported across the different density fractions within the soils, including Andisols, Spodosols, Inceptisols, Mollisols, and Ultisols (Wagai et al., 2020), which testified to the prevalence of Fe_PP_ promoting OC preservation. Historically, OC extracted by Na-pyrophosphate was expected to be the fraction of humus complexed with Al/Fe oxides (Higashi, 1983). However, the ^14^C enrichment in OC_PP_ (compared with OC_HH_ and OC_DH_) (Heckman et al., 2018), as well as the highest Fe_PP_ in a low-density fraction of soil (Wagai et al., 2020), indicated that OC_PP_ was more labile and had a faster turnover compared to the OC associated with other Fe phases.

Long-term flooding and Hem addition cause fractional crystalline Fe oxides to activate to convert to amorphous Fe oxides (Wahid and Kamalam, 1993; Rasmussen et al., 2018), whereas the capacity-stabilized OC of Fe_HH_ was considerably lower than that of Fe_PP_ (Figures 1A,2B). The molar ratios of OC and Fe oxides extracted by OC_HH_/Fe_HH_ and OC_DH_/Fe_DH_ were below 6 (Figures 2E,F), indicating that Fe-C associations were mono-layer adsorptions (Wagai and Mayer, 2007). The ultra-high OC_PP_/Fe_PP_ (Figure 2D) may reflect the fact that Fe oxides were only associated with a portion of some large organic biomolecules through only one or a few functional groups (Barber et al., 2017).

The path coefficients of Fe_PP_ to OC preservation were higher in SW than in FW (Figure 2A). This could be attributed to the relatively low rates of dissimilatory Fe reduction in SW compared to FW (Figures 2A,D), and OC stabilized by Fe oxides was released from its bound forms at relatively low levels (Pan et al., 2016; Patzner et al., 2020). Overall, our results confirmed the dominance of Fe_PP_ for OC preservation in wetlands, and we suggest that Fe_PP_ should be included in future research based on the Fe-C relationship in wetlands or water-logged soils.

### 4.2 Fe oxides regulated OC mineralization through shaping microbial community

The coupled associations of Fe-C-microbes are important for recognizing OC mineralization (Merino et al., 2021; Jeewani et al., 2021b). Consistent with the second hypothesis, Hem notable impacts OC mineralization by regulating the microbial community structure. Random forest regression showed that the bacterial phyla Actinobacteria, Bacteroidetes, and Firmicutes were the main factors influencing OC mineralization in FW, whereas fungal phyla had a very low contribution (Supplementary Figure S1a), which is consistent with the findings of previous studies, which reported that Actinobacteria and Bacteroidetes were important predictors of OC mineralization (Fierer et al., 2007; Zheng et al., 2017). Hem-treated soils had significantly higher abundance of Actinobacteria and Firmicutes than Lig-treated soils and CK (*p* < 0.05, Figure 5), which demonstrated that the fast-growing bacterial phyla preferentially colonize Fe-bearing mineral and Fe-rich soils (Converse et al., 2015; Jeewani et al., 2021b). Actinobacteria play a vital role in OC decomposition (Kopecky et al., 2011), especially polycyclic aromatic hydrocarbons, polysaccharides, and proteins (Chen et al., 2016). The lower percentage of N-containing compounds in Hem soils compared to that in others (Supplementary Figure S3D) may be attributed to the relatively high abundance of Actinobacteria because of Hem addition. The copiotrophic Actinobacteria and Bacteroidetes were abundant in OC-enriched environments, which was also observed in FW and SW soils. Generally, the enrichment of copiotrophic bacterial phyla inhibits oligotrophic bacterial phylum growth (Eo and Park, 2016; Xu et al., 2020). A low abundance of copiotrophic Chloroflexi in FW due to the formation of a copiotrophic soil environment by Lig was also observed in our study (Figure 5C).

Our results showed that the DIR of Hem was lower than that of Lig in FW (Figure 3A), which can be attributed to the reduction pathway of Fe-reducing bacteria, initial soil pH, and the toxicity of Fe oxide nanoparticles. First, the dominant Fe-reducing bacteria in Lig-treated soils included Anaeromyxobacter, Bacillus, and Clostridium, and Hem-treated soils were enriched with Bacillus and Clostridium (Fermentative Fe reducers), whereas Lig induced a number of Fe respiration reducers (e.g., Anaeromyxobacter and Geobacter; Figure 6) (Lentini et al., 2012). Theoretically, 1 mol of glucose thoroughly oxidized to CO_2_ by Geobacter would produce 24 mol of Fe(II). Therefore, Fe(III) reducers that had higher potential for Fe(III) reduction in Lig were responsible for higher rates of DIR (compared to that in Hem soils). Second, Lig or Hem could decrease the pH, which would influence the DIR in FW. The optimum pH for Geobacter and Anaeromyxobacter is 6.7–7.0 (Porsch et al., 2009; Hori et al., 2010), and an initial pH <6.0 in FW soil inhibited the DIR rate (Blöthe et al., 2008). The pH decrements caused by Hem and/or Lig further suppressed the growth of Fe(III) reducers (Rousk et al., 2010). However, more protons were consumed with DIR processing, and the pH increased. Third, the toxicity of the Fe oxide nanoparticles (Hem) inhibited the growth of Fe-reducing bacteria and further decreased the rate of DIR. Possible mechanisms may include interference in the cellular uptake of nutrients and cell destruction (Yuan et al., 2021).

Hem-treated FW soils showed lower DIR rates than Lig but yielded the highest cumulative carbon emissions. This phenomenon indicated that other anaerobic OC decomposition pathways were activated by Hem, such as anaerobic methane oxidation mediated by Fe(III) (Yan et al., 2018; He et al., 2019). Overall, this study demonstrated that (1) the alteration of the microbial community by Hem and Lig determined the anaerobic OC mineralization; and (2) the rate of DIR may be determined by the reduction pathway of Fe-reducing bacteria, initial pH, and the toxicity of Fe oxide nanoparticles. Furthermore, considering the wide distribution of fermentative Fe reducers, their relationship with OC-Fe needs more attention.

### 4.3 The balance of Fe-mediated OC preservation and mineralization in wetlands

We only partially accepted our hypothesis owing to the negative effect of Hem on OC accumulation under anaerobic conditions. Hem not only decreased OC stabilized by Fe oxides but also increased Fe-mediated OC mineralization, such as DIR and Fe(III)-mediated anaerobic ammonium oxidation, by shaping microbial communities. This finding is consistent with increased CO_2_ emissions in anoxic conditions because of microbial Fe(III) reduction and decreased CH_4_ emissions suppressed by Fe(II) (Figure 3), which can be attributed to the following reasons: a long-term flooding environment facilitated the mobilization of soil crystalline Fe oxides and exogenous Hem to amorphous Fe oxides and provided more electronic acceptors for microbial respiration. However, Hem substantially shifts the microbial community structure and shapes metabolic traits (Jeewani et al., 2021a,b). Although Lig induced native SOC decomposition, this effect was lower than the preservation effect through the formation of Fe-C complexes. According to the CO_2_ and CH_4_ emission rates (Figure 3), we only found considerable OC mineralization rates on the first 4 days, which decreased in the following incubation days, indicating that the labile fraction of Lig was quickly utilized by microorganisms and the relative recalcitrant fraction was bound to Fe oxides, contributing to OC persistence (Schmidt et al., 2011; Lehmann and Kleber, 2015).

Overall, OC-Fe may not be a stable reservoir for long-term SOC storage, but this carbon pool is dynamic (Keiluweit et al., 2015). Moreover, considering the redox sensitivity of Fe oxides, further studies should assess the net effect of Fe oxide-mediated OC preservation and mineralization in redox-dynamic soils. Additionally, further research should focus on the double effect on OC caused by reactive Fe oxides in anaerobic or redox-dynamic conditions and the fermentative Fe reducer in the coupled C-Fe relationship.

## 5 Conclusion

We found that Hem did not facilitate OC preservation by Fe oxides, and Lig improved OC stability by increasing the coupled OC-Fe association. Hem introduction inhibited microbe-mediated Fe(III) reduction of FW by decreasing the relative abundance of respiratory Fe reducers (Geobacter and Anaeromyxobacter) but dramatically enhanced OC mineralization by shaping microbial communities, e.g., by supporting the high relative abundance of Actinobacteria and Firmicutes. Lig resulted in the highest cumulative carbon emissions in SW, and it induced the intensity of preservation through the formation of organo-mineral complexes (which was far higher than mineralization). These results could be valuable in wetland restoration applications, for example, it is good to add aromatic rich carbon to associate with Fe oxides and reduce the Fe induced carbon mineralization during wetland recovery, which would be useful method to promote carbon sink during wetland recovery.

## Data availability statement

The original contributions presented in the study are included in the article/Supplementary material, further inquiries can be directed to the corresponding authors.

## Author contributions

BN: Investigation, Writing – original draft, Formal analysis. XY: Writing – review & editing, Investigation, Methodology. XD: Writing – original draft, Methodology, Writing – review & editing. YZ: Writing – original draft, Funding acquisition, Methodology, Writing – review & editing.

## Footnotes

^1^ https://www.r-project.org
