# Supplementary material for: Wetland soil organic carbon balance is reversed by old carbon and iron oxide additions
Source: Front Microbiol. 2024 Jan 8;14:1327265. doi: 10.3389/fmicb.2023.1327265 (PMC10800826; doi:10.3389/fmicb.2023.1327265)
Supplement: Supplementary file 1 [file Data_Sheet_1.docx]

Supplementary Material

## Supplementary Figures


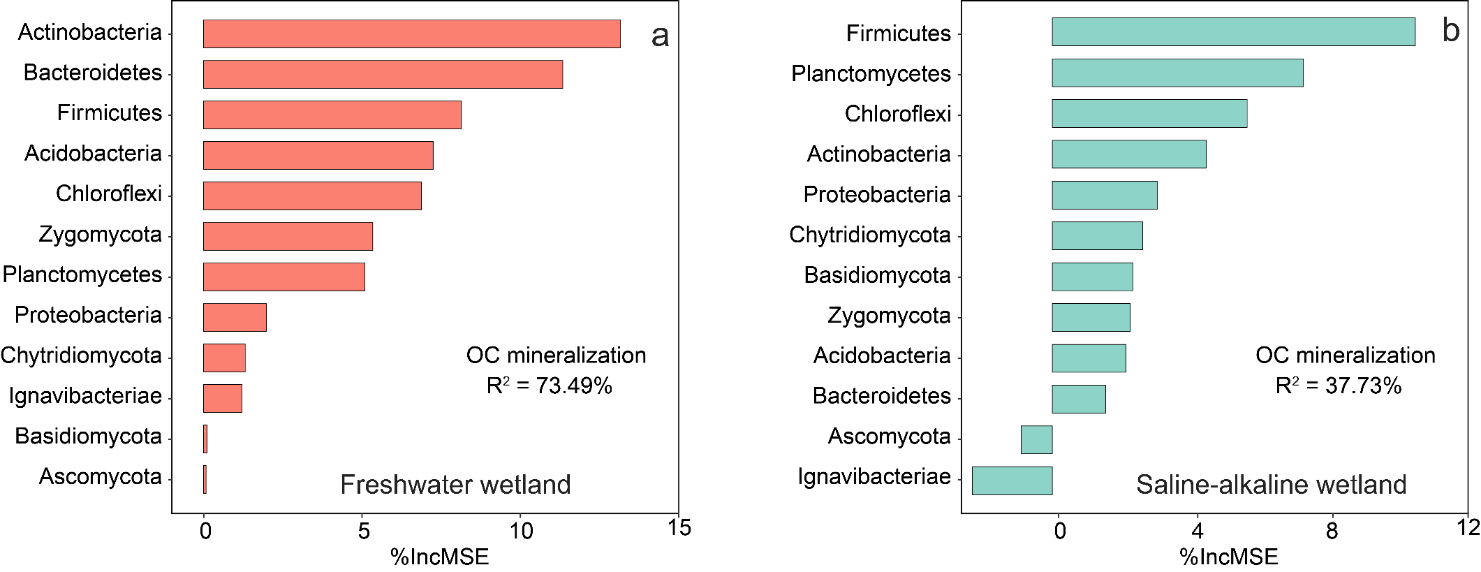


**Fig. S1.** Relative importance of bacterial and fungal phyla for the explanation of OC stabilization and mineralization of freshwater (a, c) and saline-alkaline (b, d) wetlands soils after 56 days of anaerobic incubation performed by random forest analysis (n = 6). The relative importance was represented by the value of the percentage increase in mean square error (%IncMSE). R2 represented the proportion of the variance for OC stabilization and mineralization of freshwater and saline-alkaline (b, d) wetlands soils.


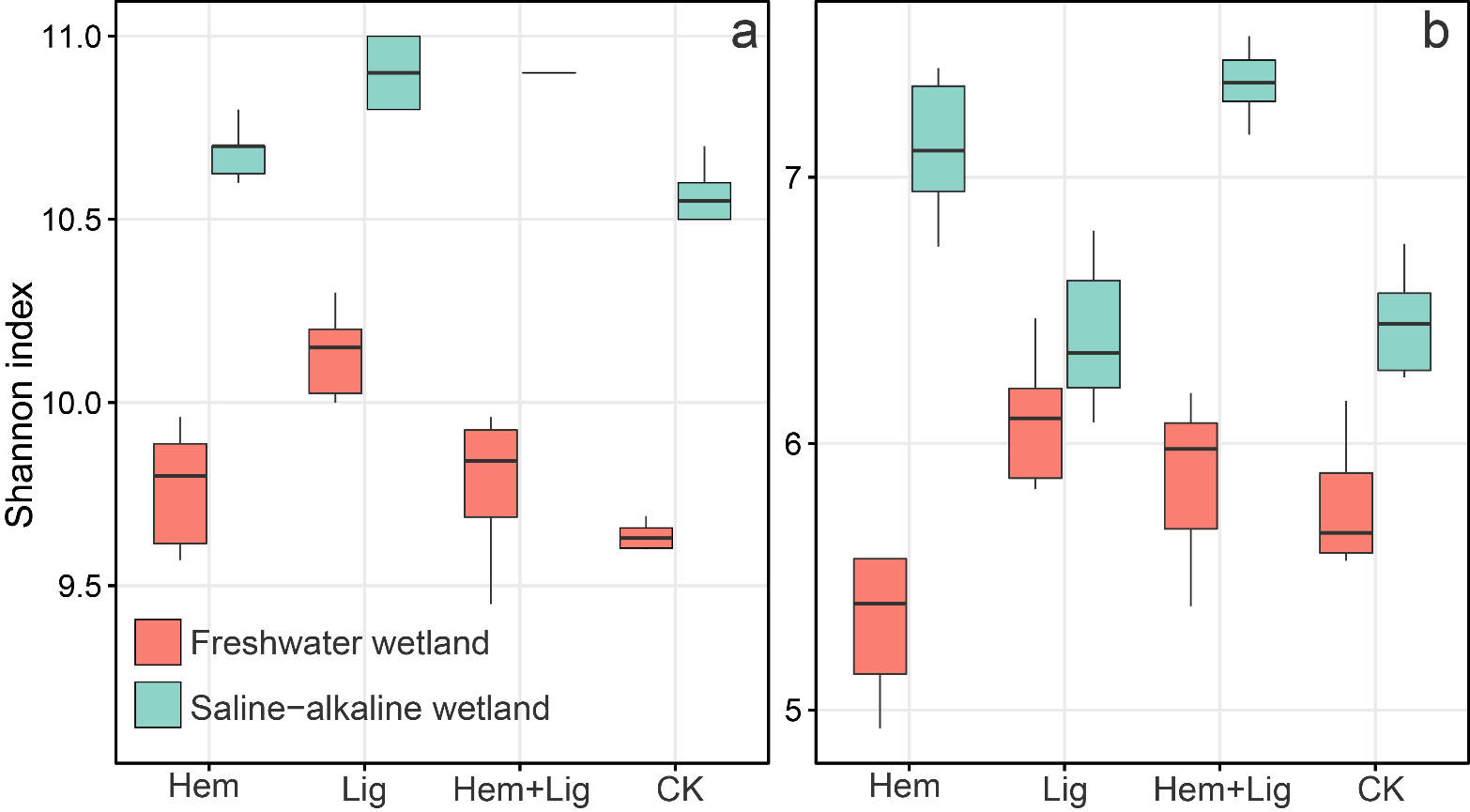


**Fig. S2.** Shannon index of bacteria (a) and fungi (b) of freshwater (red boxes) and saline-alkaline (green boxes) wetlands soils responding to Hem and Lig after 56 days of anaerobic incubation (n = 6).


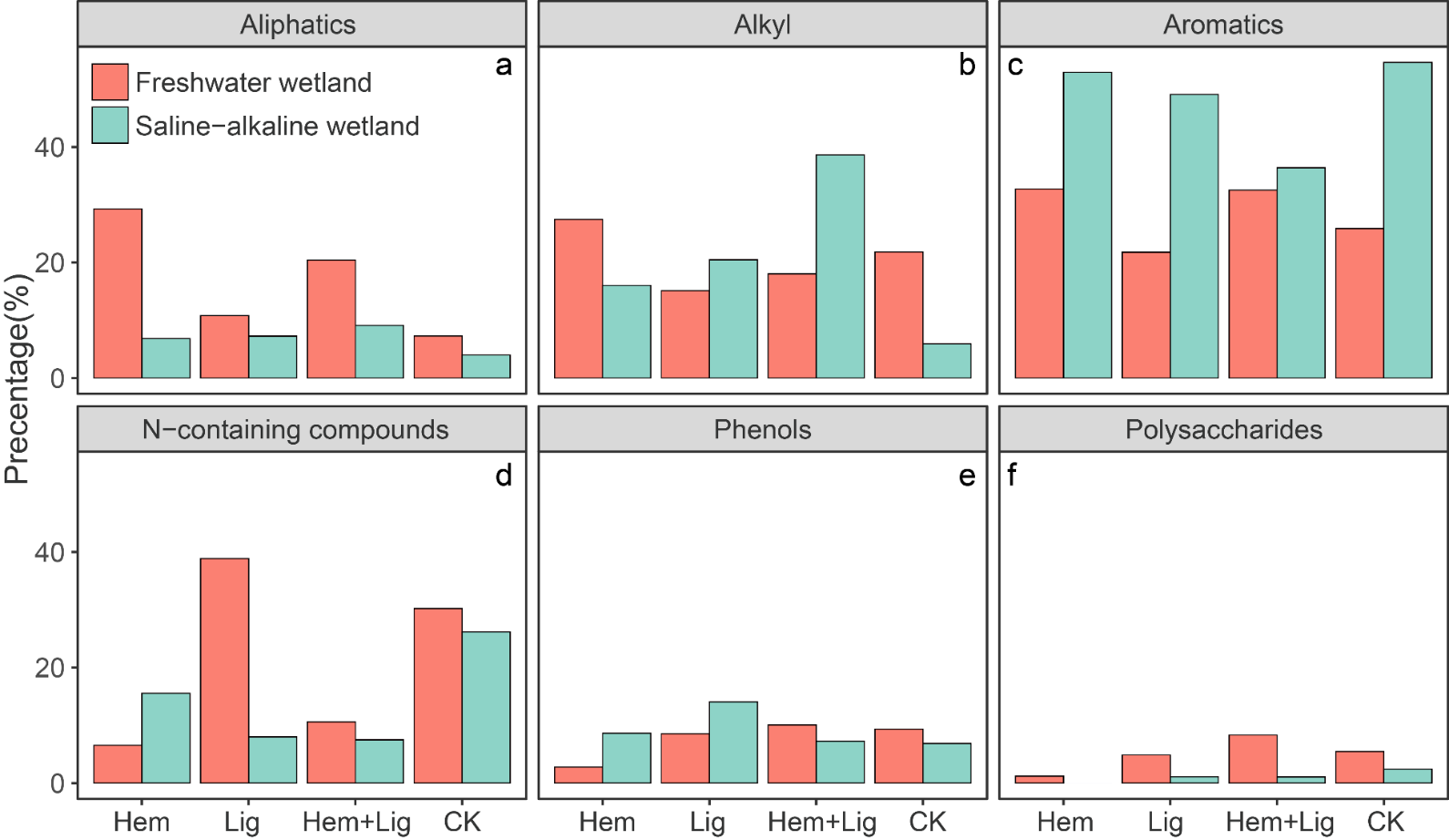


**Fig. S3.** Pyrolysis-gas chromatography/mass spectrometry (Py-GC/MS) showed soil organic carbon chemical composition including aliphatic (a), alkyl (b), aromatics (c), N-containing compounds (d), phenols (e), and polysaccharides (f) in freshwater and saline-alkaline wetlands soils response to hematite (Hem) and lignite (Lig) addition.
